# Dietary Supplementation with L-Serine Regulates Growth Performance, Carcass Traits, Meat Quality, and Intestinal Development in Broilers

**DOI:** 10.3390/ani15202965

**Published:** 2025-10-13

**Authors:** Longlong Li, Mei Su, Lan Zhang, Junyi Luo, Ting Chen, Jiajie Sun, Yongliang Zhang, Qianyun Xi

**Affiliations:** Guangdong Provincial Key Laboratory of Animal Nutrition Control, National Engineering Research Center for Breeding Swine Industry, College of Animal Science, South China Agricultural University, No. 483 Wushan Road, Guangzhou 510642, China

**Keywords:** L-serine, broiler, growth performance, meat quality, intestinal development

## Abstract

**Simple Summary:**

This study evaluated the effects of adding L-serine to broiler diets. Appropriate supplementation significantly improved growth performance and feed conversion efficiency, reduced localized fat deposition, enhanced meat quality, and promoted intestinal morphological development. As a safe, residue-free feed additive, L-serine has the potential to increase broiler production efficiency and help ensure meat safety.

**Abstract:**

This study evaluated the effects of additional L-serine supplementation in diets on performance, carcass characteristics, meat quality and small intestine development in broilers. A total of 300 one-day-old unsexed Yellow-Feathered broilers were randomly divided into three groups with 10 replicates of 10 birds each. The broilers were fed basal diet (CON), basal diet + 0.085% L-Alanine (Isonitrogenous) and basal diet + 0.1% L-serine (0.1% Ser), respectively. The whole trial lasted for 42 d. The results showed that 0.1% L-serine supplementation in broiler diets could increase final body weight and average daily weight gain (*p* < 0.05) and had no significant effect on serum biochemical indexes (*p* > 0.05). Compared with the Isonitrogenous group, the 0.1% Ser group significantly reduced FCR (*p* < 0.05). Regarding meat quality, the 0.1% Ser group significantly increased by a*45 min and 24 h pH values, while it decreased by b*45 min values in both breast and leg muscles (*p* < 0.05) and reduced cooking loss in leg muscles (*p* < 0.05). L-serine effectively reduced localized fat deposition and promoted intestinal development. Morphometric analysis revealed significantly increased small intestinal villus length and villus length/crypt depth ratio in the 0.1% Ser group (*p* < 0.05). In conclusion, L-serine can be used as an effective supplement in broiler farming to improve its productivity.

## 1. Introduction

Chicken has become an essential meat in the human diet due to its low cost and high nutritional properties. However, various factors in intensive farming environments often lead to imbalance of homeostasis and increased oxidative stress in broilers [1], which results in reduced performance and meat quality. And with the increase in global production and consumption of poultry meat, the poultry industry is faced with a huge challenge of maintaining the health and well-being of poultry [2]. Many different feed additives are frequently used in modern poultry production to maintain good health and metabolic status and to improve performance indicators of intensively farmed animals. Most sensory, processing, animal production technology, medicinal, and nutritional feed additives enhance feed characteristics, animal health, or production efficiency. Notably, nutritional additives possess unique properties, supplying essential nutrients (such as amino acids like L-serine, vitamins, and minerals) required to maintain fundamental physiological functions and metabolic pathways. Exploring the discovery of feed additives with no residues, no toxic side effects and no resistance is important for the development of animal husbandry and ensuring human health and safety [3,4,5].

L-serine, as a non-essential amino acid, is located in the central node of body metabolism, and it can regulate the body through various metabolic pathways [6]. For example, L-serine plays a key role in effector T cell responses through one-carbon metabolism [7], and it can also directly regulate adaptive immunity by controlling T cell proliferation [8]. Serine deficiency exacerbates oxidative stress and inhibits enterocyte proliferation, which is reversed by exogenous serine supplementation [9]. In addition, serine alleviates oxidative stress in the hepatic by supporting glutathione synthesis and methionine cycling [10]. In terms of intestinal inflammation and intestinal microbiota, serine also exerts a positive influence [11]. As for poultry, L-serine serves as a critical glycine equivalent (Glyequi) in poultry nutrition, enabling significant crude protein reduction (to 15–16% in starter diets) without compromising growth performance by optimizing nitrogen utilization for uric acid synthesis and reducing urinary nitrogen excretion by ≥25% [12]. Additional supplementation of L-serine in broiler rations to counteract the hazards associated with intensive feeding may be a viable tool.

The study aimed to evaluate effects of L-serine on growth performance, carcass traits, meat quality, and intestinal development in broilers.

## 2. Materials and Methods

### 2.1. Animal Ethics Statement

The experiment was approved by the Animal Care Institution and Ethics Committee of South China Agriculture University. All animal experiments were accepted by the Animal Ethics Committee of South China Agricultural University, with permit number SYXK (Guangdong) 2022-0136 (13 January 2022).

### 2.2. Experimental Design and Bird Management

Three dietary treatments with ten replicate groups (ten broilers were fed in each replicate) were randomly assigned to 300 one-day-old, unsexed Yellow-Feathered broilers (Hongji Company of Guangzhou, China) with similar body weights: CON group (basal diet), Isonitrogenous group (basal diet + 0.085% L-Alanine), and 0.1% Ser group (basal diet + 0.1% L-Serine). L-Serine and L-Alanine from Xiangsheng Company of Yancheng, China. The broilers were unsexed, with an average initial body weight of about 39 g, and were well-born and healthy. Birds were vaccinated according to standardized procedures at hatch. Subsequently, broilers were kept in stainless-steel cages (85 cm × 80 cm × 35 cm) at a density of five birds per cage, with free access to water and feed throughout the trial. The temperature was kept at 34 °C (1–7 days), subsequently gradually reduced to 20–21 °C at a rate of 2–3 °C per week. Feed intake, body weight, average daily feed intake (ADFI), average daily gain (ADG), and feed/gain ratio (FCR) were all recorded weekly for each cage during the 42-day study. The basal diet was formulated following the NRC (1994) standard and China’s “Chicken Feeding Standard (NY/T33-2004)” (issued by the Ministry of Agriculture) to meet nutritional requirements. Table 1 displays the nutritional level and content of baseline diets.

### 2.3. Serum Biochemical Indices

At the termination of the trial, blood samples were collected from the broiler’s wing veins and centrifuged at 3000 rpm for 15 min. Following centrifugation, serum was retrieved from the supernatant, moved to sterile fresh tubes, and kept at −20 °C until analysis. The automatic biochemical analyzer (Model 7600 Series Automatic Analyzer, Hitachi, Tokyo, Japan) was used to measure the serum biochemical indices (TP, total protein; GLB, globulin; ALB, albumin; TG, triglyceride; GLU, glucose; TCH, total cholesterol; UA, uric acid; LDL-CH, low-density lipoprotein cholesterol; HDL-CH, high-density lipoprotein cholesterol).

### 2.4. Carcass Trait Measurement

Upon completion of the experiment, one broiler was randomly selected from each replicate after 12 h of fasting, then put to death by cervical dislocation following electrical stunning at 50 V. The body weight after removal of the internal organs, limbs, and abdominal fat was recorded as eviscerated yield. Then, the abdominal fat, bilateral breast muscles, and leg muscles were weighed, and the ratio of abdominal fat, breast muscles, and leg muscles weight relative to slaughter weight was computed. Subsequently, measurements of intermuscular fat breadth and subcutaneous fat depth were conducted.

### 2.5. Meat Quality Parameter Measurement

Using the techniques of earlier research, the color, pH, shear value, cooking loss, and drip loss of the breast and leg muscles were assessed [13].

### 2.6. Small Intestine Developmental Measurement

The duodenum, jejunum, and ileum were carefully separated at the time of slaughter, and the length of each segment was accurately measured with a straightedge and weighed to calculate the relative intestinal length (cm/kg, intestinal length/broiler weight) and relative intestinal weight (%, intestinal weight/broiler weight). In addition, 2 cm tissue samples were taken from the duodenum, jejunum and ileum at midpoint for HE staining. The intestinal morphology was observed under light microscope, and villus height, crypt depth and V/C (villus height/crypt depth) values were counted.

### 2.7. Statistical Analysis

One-way ANOVA and the Tukey post hoc test were used to analyze the data using SPSS 25.0. Every data point is displayed as mean ± SEM. The threshold for statistical significance was set at *p* < 0.05.

## 3. Results

### 3.1. Growth Performance and Serum Biochemical Indicators

The effect of dietary serine on the growth performance of broiler chickens is presented in Table 2. Compared with the Isonitrogenous group, the Final weight and ADG in 0.1% Ser groups increased significantly (*p* < 0.05), while the FCR decreased significantly (*p* < 0.05). However, the FCR of broiler chickens in the CON group did not differ significantly from that of the 0.1% Ser group (*p* > 0.05). There was no significant difference in ADFI of all treatment groups (*p* > 0.05). Serum biochemical indicators across all treatment groups were assessed (Table 3), and the results showed that serum TP, GLB, ALB, GLU, TG, TCH, UA, LDL-CH, and HDL-CH were not significantly different among the groups (*p* > 0.05).

### 3.2. Carcass Traits

The carcass traits of broiler chickens are shown in Table 4; only intermuscular fat produced changes after treatment. Compared to the CON and isonitrogenous groups, the 01% Ser group significantly reduced intermuscular fat content (*p* < 0.05).

### 3.3. Meat Quality

Breast and leg muscle meat quality parameters were evaluated separately. As shown in Table 5, the a*45 min and 24 h pH values of breast muscle in the 0.1% Ser group were significantly higher than those in the CON and Isonitrogenous groups, while the opposite was true for the b*45 min values (*p* < 0.05). Meanwhile, there was no difference in drip loss, cooking loss and shear value of pectoral muscle in all treatment groups (*p* > 0.05). While the results for the leg muscles are shown in Table 6, similar to the results for the breast muscles, the a*45 min value and 24 h pH value of the leg muscles in the 0.1% Ser group were increased, while the b*45 min value was decreased (*p* < 0.05). In addition, the 0.1% Ser group reduced cooking losses in the leg muscles (*p* < 0.05).

### 3.4. Intestinal Development

**Figure 1:**
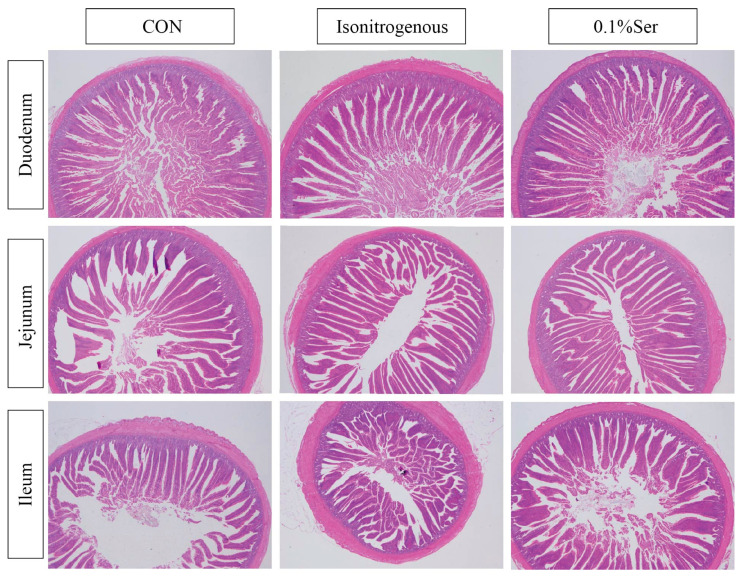
Effect of dietary 0.1% Ser on intestinal mucosal morphology.

As shown in Table 7 and Figure 1, serine treatment had a positive effect on all intestinal segments. Relative weights of duodenum, jejunum and ileum were increased in the 0.1% Ser group compared to the other groups (*p* < 0.05), whereas only duodenum produced significant differences in relative length (*p* < 0.05), with no significant differences in the relative length of the jejunum and ileum (*p* > 0.05). For intestinal morphology, the 0.1% Ser group increased the villus height and V/C ratio of the duodenum, jejunum and ileum (*p* < 0.05). At the same time, crypt depth in the duodenum and ileum of the 0.1% Ser group was significantly reduced compared to the isonitrogenous group (*p* < 0.05). There was no significant difference in the crypt depth of ileum among the treatment groups (*p* > 0.05).

## 4. Discussion

Non-essential amino acids play important roles in protein synthesis, hormone secretion, intestinal integrity, antioxidant defense, and immunity [14]. Therefore, additional supplementation of animals’ diets with appropriate amounts of non-essential amino acids can optimize their survival, growth, developmental reproduction, and health. The addition of both glycine and serine to low-protein diets has been shown to improve broiler performance [15]. Serine also partially replaces the need for glycine in chicks, and serine plays a key role in maintaining optimal performance in broilers [16]. Dietary supplementation with 0.1% L-serine significantly increased final body weight and ADG (*p* < 0.05), aligning with previously reported growth responses [17]. However, in our trial we found that FCR was significantly lower in the 0.1% L-serine group compared to the isonitrogenous group (*p* < 0.05). As for the serum biochemical indices, additional L-serine supplementation did not affect serum levels of TP, GLB, ALB, TG, GLU, TCH, UA, LDL-CH, and HDL-CH in broilers.

Serine is involved in the regulation of protein synthesis and lipid metabolism in the body [18]. Previous studies have shown that excessive fat deposition is detrimental to the economic value of broilers and that serine has the potential to reduce fat deposition in broilers [17,19]. L-serine provides an essential nitrogen source for broilers during the growing period, promotes protein synthesis in the body, and mobilizes nutrient partitioning in the body resulting in reduced fat deposition. In our experiment, the addition of 0.1% L-serine to the diet significantly (*p* < 0.05) reduced intermuscular fat width in broilers compared to other groups. Although it did not reach significance, abdominal fat percentage and subcutaneous fat percentage were reduced, suggesting that L-serine has the potential to improve carcass quality in broilers.

pH is one of the most important parameters of meat quality as it is positively correlated with meat water holding capacity, redness and tenderness [20] and negatively correlated with meat brightness [21] and drip loss [22]. In broilers, lactic acid and anaerobic glycolysis accumulate in the muscle at about 24 h post-slaughter, and the pH drops rapidly, which leads to denaturation of proteins, which in turn affects the color and water retention capacity of the meat [23]. In this experiment, the addition of L-serine significantly increased the pH of pectoral and leg muscles for 24 h, and also reduced the cooking loss of leg muscles. Meat color was reflected by brightness L*, red color a* and yellow color b*. The results of this experiment showed that L-serine significantly increased the a* values of pectoral and hamstrings muscles while decreasing the b*. In addition, the L* of the hamstrings was significantly reduced. It has been reported that serine blocked the lipopolysaccharide-induced increase in hepatic superoxide radical, hydrogen peroxide, and TBARS levels and restored the Diquat treatment-induced decreases in GSH levels, GSH/GSSG ratio, and GSH-Px activity [10]. In the present study, L-serine may promote muscle antioxidant properties and affect muscle myoglobin content by increasing antioxidant enzyme activities, inhibiting lipid peroxidation and peroxide accumulation, and thus presenting higher a* in pectoral and leg muscles. Increasing the antioxidant capacity of muscle would be beneficial to the meat quality of chicken during transportation and preservation. Therefore, L-serine can be used as an effective additional supplement to improve the quality of chicken meat.

As a direct result of the small intestine’s morphological and function development, chicken growth is dependent on the nutritional digestion and uptake that it affects. Characteristics of the digestive tract affect the efficiency of diet utilization, and, in particular, the microstructure of the small intestine in terms of villus height and crypt depth are considered to be the main indicators of intestinal development, health and function, affecting the digestion and absorption of nutrients [24]. Shortened villi and deeper crypts can lead to malabsorption of nutrients, increased intestinal secretion and decreased performance [25]. Body weight gain is associated with increased villus height, villus size and cell mitosis in chickens [26]. Recent studies have shown that the addition of serine improves growth performance and intestinal development in piglets through tight junction protein synthesis and attenuation of apoptosis and oxidative stress in the intestine [27]. In addition, glycine also promotes proliferation of small intestinal cells and plays an important role in regulating cell growth [28]. Serine is known to convert glycine with an efficiency of 71.43%; thus, serine acts as a precursor to glycine [15], and it is hypothesized that serine may enhance intestinal function through conversion to glycine. The promotional effect of L-serine on intestinal development was further confirmed in our results, where the relative intestinal weights of all parts of the small intestine were significantly increased by L-serine supplementation, and the relative length of the duodenum was also enhanced compared to the other groups. Meanwhile, similar to the results of previous studies [17], histological examination using HE staining revealed that L-serine treatment significantly increased villus length and the villus height/crypt depth ratio in all intestinal segments. Concurrently, crypt depth in the duodenum and jejunum was markedly reduced compared to the control group. Combined with the results of growth performance, it is hypothesized that L-serine mainly promotes intestinal development to increase the absorption of food in the intestinal region, which facilitates the digestion and absorption of food in the intestinal tract and thus promotes the growth of broilers.

## 5. Conclusions

The results of the current study indicate that supplementation of broiler diets with 0.1% L-serine improves growth performance, carcass traits and meat quality. In addition, L-serine promotes the development of the small intestine in broilers, which facilitates the digestion and absorption of nutrients in the small intestine. Additional L-serine can be an effective means of increasing broiler productivity.

## Figures and Tables

**Table 1:** Composition and nutrient levels of broiler basal diets at various stages (%).

Items	1–21 Days of Age	22–42 Days of Age
Corn	60.43	65.32
Soybean meal	33.50	27.49
Corn protein powder	0.00	1.00
Soybean oil	1.50	2.00
L-lysine HCl	0.00	0.03
DL-Methionine	0.22	0.11
Calcium hydrogen phosphate	2.16	1.55
Limestone	0.89	1.20
Salt	0.30	0.30
Premix ^1^	1.00	1.00
Nutrient level ^2^		
ME (MJ/kg)	12.22	12.55
Crude protein	21.00	19.00
Calcium	0.95	0.90
Total phosphorus	0.76	0.64
Available phosphorus	0.54	0.40
Lysine	1.13	1.00
Methionine + Cystine	0.87	0.71

^1^ Premix supplied per kg of diet: vitamin A, 15,000 IU, vitamin D_3_, 3300 IU, vitamin E, 10 IU, vitamin K_3_, 0.5 mg, vitamin B_1_, 3.8 mg, vitamin B_2_, 4.0 mg, vitamin B_3_, 42 mg; choline, 600 mg; vitamin B_5_, 10 mg; vitamin B_6_, 3.5 mg vitamin B_12_, 0.01 mg, biotin, 0.15 mg, folic acid, 0.55 mg, Fe, 80 mg, Cu, 8 mg, Zn, 75 mg, Mn, 60 mg, Se, 0.15 mg, I, 0.35 mg. ^2^ Except for crude protein and metabolic energy levels, all other values were calculated.

**Table 2:** Effect of serine on the growth performance of broilers.

Items ^1^	Groups ^2^			SEM ^3^	*p*-Value
	CON	Isonitrogenous	0.1%Ser		
Initial weight (g)	39.21	39.71	38.85	0.27	0.45
Final weight (g)	1135.61 ^b^	1146.35 ^b^	1200.36 ^a^	11.72	<0.05
ADG (g)	26.10 ^b^	26.35 ^b^	27.66 ^a^	0.28	<0.05
ADFI (g)	46.62	47.78	47.24	0.32	0.35
FCR	1.79 ^ab^	1.81 ^a^	1.71 ^b^	0.02	<0.05

^a,b^ Means within a row with different superscripts are different at *p* < 0.05. ^1^ ADG, average daily gain; ADFI, average daily feed intake; FCR, feed conversion ratio (feed:gain, g:g). ^2^ CON, basal diet; Isonitrogenous, basal diet + 0.085% L-Alanine; 0.1% Ser, basal diet + 0.1% L-Serine. ^3^ SEM, standard error of the mean (*n* = 10).

**Table 3:** Effect of serine on serum biochemical indicators of broilers.

Items ^1^	Groups ^2^			SEM ^3^	*p*-Value
	CON	Isonitrogenous	0.1%Ser		
TP (g/L)	36.0	32.6	33.3	0.91	0.36
GLB (g/L)	14.1	12.5	12.7	0.35	0.21
ALB (g/L)	21.9	20.1	20.7	0.64	0.56
GLU (mmol/L)	13.1	13.8	13.1	0.29	0.57
TG (mmol/L)	0.3	0.3	0.3	0.01	0.48
TCH (mmol/L)	3.4	3.1	3.3	0.09	0.33
UA (umol/L)	177.3	174.7	174.3	7.20	0.99
LDL-CH (mmol/L)	0.9	0.9	1.0	0.04	0.80
HDL-CH (mmol/L)	2.2	2.1	2.3	0.05	0.50

^1^ TP, total protein; GLB, globulin; ALB, albumin; TG, triglyceride; GLU, glucose; TCH, total cholesterol; UA, uric acid; LDL-CH, low-density lipoprotein cholesterol; HDL-CH, high-density lipoprotein cholesterol. ^2^ CON, basal diet; Isonitrogenous, basal diet + 0.085% L-Alanine; 0.1% Ser, basal diet + 0.1% L-Serine. ^3^ SEM, standard error of the mean (*n* = 10).

**Table 4:** Effect of serine on carcass traits of broilers.

Items	Groups ^1^			SEM ^2^	*p*-Value
	CON	Isonitrogenous	0.1%Ser		
Dressing percentage (%)	90.51	90.03	90.91	0.28	0.42
Eviscerated yield with giblet (%)	82.91	83.74	83.69	0.79	0.87
Eviscerated yield (%)	68.69	68.69	67.32	0.58	0.55
Breast muscle yield (%)	15.09	15.83	14.97	0.30	0.45
Leg muscle yield (%)	19.37	18.76	19.99	0.43	0.50
Abdominal fat (%)	2.00	2.50	1.83	0.20	0.39
Subcutaneous fat depth (mm)	2.00	2.06	1.87	0.08	0.61
Intermuscular fat width (mm)	6.01 ^a^	5.57 ^a^	4.38 ^b^	0.25	<0.01

^a,b^ Means within a row with different superscripts are different at *p* < 0.05. ^1^ CON, basal diet; Isonitrogenous, basal diet + 0.085% L-Alanine; 0.1% Ser, basal diet + 0.1% L-Serine. ^2^ SEM, standard error of the mean (n = 10).

**Table 5:** Effect of serine on meat quality of broiler breast muscle.

Items ^1^	Groups ^2^			SEM ^3^	*p*-Value
	CON	Isonitrogenous	0.1%Ser		
L*45 min	51.31	50.78	52.61	0.52	0.35
a*45 min	5.06 ^b^	4.86 ^b^	9.03 ^a^	0.44	<0.01
b*45 min	7.62 ^a^	8.18 ^a^	4.83 ^b^	0.36	<0.01
pH 45 min	6.29	6.34	6.21	0.04	0.89
pH 24 h	5.65 ^b^	5.70 ^b^	5.79 ^a^	0.02	<0.01
Drip loss (%)	1.71	1.92	1.79	0.07	0.58
Cooking loss (%)	24.26	23.87	25.31	0.62	0.65
Shear value (N)	10.50	9.99	9.98	0.32	0.77

^a,b^ Means within a row with different superscripts are different at *p* < 0.05. ^1^ a*, redness; b*, yellowness; L*: luminance. ^2^ CON, basal diet; Isonitrogenous, basal diet + 0.085% L-Alanine; 0.1% Ser, basal diet + 0.1% L-Serine. ^3^ SEM, standard error of the mean (*n* = 10).

**Table 6:** Effect of serine on meat quality of broiler leg muscle.

Items ^1^	Groups ^2^			SEM ^3^	*p*-Value
	CON	Isonitrogenous	0.1%Ser		
L*45 min	58.60 ^a^	59.03 ^a^	47.24 ^b^	1.21	< 0.01
a*45 min	8.99 ^b^	8.94 ^b^	10.74 ^a^	0.35	< 0.05
b*45 min	8.92 ^a^	6.84 ^b^	6.22 ^b^	0.35	< 0.01
pH 45 min	6.65	6.75	6.57	0.03	0.24
pH 24 h	6.11 ^b^	6.18 ^b^	6.25 ^a^	0.02	< 0.01
Drip loss (%)	2.38	2.11	2.15	0.13	0.71
Cooking loss (%)	25.26 ^a^	26.80 ^a^	18.39 ^b^	1.29	< 0.01
Shear value (N)	10.38	10.99	11.12	0.31	0.61

^a,b^ Means within a row with different superscripts are different at *p* < 0.05. ^1^ a*, redness; b*, yellowness; L*: luminance. ^2^ CON, basal diet; Isonitrogenous, basal diet + 0.085% L-Alanine; 0.1% Ser, basal diet + 0.1% L-Serine. ^3^ SEM, standard error of the mean (*n* = 10).

**Table 7:** Effect of serine on Intestinal development of broilers.

Items ^1^	Groups ^2^			SEM ^3^	*p*-Value
	CON	Isonitrogenous	0.1%Ser		
Duodenum					
relative intestinal length (cm/kg)	26.3 ^b^	25.6 ^b^	31.1 ^a^	0.92	<0.01
relative intestinal weight (%)	0.9 ^b^	1.0 ^b^	1.2 ^a^	0.04	0.01
Villus height	1573 ^b^	1653 ^b^	1943 ^a^	49.96	<0.01
Crypt depth	250 ^a b^	264 ^a^	235 ^b^	8.16	0.01
V/C	6.3 ^b^	6.1 ^b^	8.3 ^a^	0.29	<0.01
Jejunum					
relative intestinal length (cm/kg)	46.5	43.7	48.4	1.19	0.28
relative intestinal weight (%)	1.4 ^b^	1.4 ^b^	1.8 ^a^	0.08	<0.01
Villus height	1401 ^b^	1418 ^b^	1591 ^a^	15.97	<0.01
Crypt depth	222 ^a^	231 ^a^	192 ^b^	4.67	<0.01
V/C	6.3 ^b^	6.2 ^b^	8.3 ^a^	0.26	<0.01
Ileum					
relative intestinal length (cm/kg)	45.7	44.9	49.9	1.29	0.27
relative intestinal weight (%)	1.0 ^b^	1.0 ^b^	1.3 ^a^	0.05	<0.01
Villus height	1175 ^b^	1254 ^b^	1386 ^a^	31.07	<0.01
Crypt depth	191	184	171	13.06	0.31
V/C	6.3 ^b^	6.8 ^a b^	8.3 ^a^	0.63	0.02

^a,b^ Means within a row with different superscripts are different at *p* < 0.05. ^1^ Relative intestinal length, intestinal length/broiler weight; relative intestinal weight, intestinal weight/broiler weight; V/C, villus height/crypt depth. ^2^ CON, basal diet; Isonitrogenous, basal diet + 0.085% L-Alanine; 0.1% Ser, basal diet + 0.1% L-Serine. ^3^ SEM, standard error of the mean (*n* = 10).

## Data Availability

The data presented in this study are available upon request from the corresponding author.
